# Effect of nutrition intervention on cognitive development among malnourished preschool children: randomized controlled trial

**DOI:** 10.1038/s41598-023-36841-7

**Published:** 2023-06-30

**Authors:** Ansuya, Baby S. Nayak, B. Unnikrishnan, Y. N. Shashidhara, Suneel C. Mundkur

**Affiliations:** 1 Department of Community Health Nursing, Manipal College of Nursing, Manipal Academy of Higher Education, Manipal, Karnataka India; 2 Department of Child Health Nursing, Manipal College of Nursing, Manipal Academy of Higher Education, Manipal, Karnataka India; 3 Kasturba Medical College, Manipal Academy of Higher Education, Mangaluru, Karnataka India; 4 Department of Pediatrics, Kasturba Medical College, Manipal Academy of Higher Education, Manipal, Karnataka India

**Keywords:** Health care, Signs and symptoms

## Abstract

Childhood malnutrition impairs health, development, and productivity in adulthood. Underweight children have been found to have a variety of cognitive abnormalities. The present study examined the effect of a nutrition-focused intervention on cognitive development among malnourished preschool children between 3 and 5 years of age residing in selected villages of Udupi district, Karnataka. A cluster of 12 villages was chosen randomly. The trial had enrolled preschool children (n = 253) from randomly assigned selected villages to intervention (n = 127) and control arms (n = 126). The mothers in the intervention arm received nutrition-focused intervention and reinforcement of health teaching for 12 months. The post-intervention outcome on the cognitive development of malnourished children was measured at 6 months and 12 months. Statistical analyses indicated that 52% of children in the intervention group had average cognitive development scores on the pre-test, whereas on the post-test, only 5.5% were in the average level of cognitive development. In the control group, the average cognitive development status of the children decreased from 44.4% in the pretest to 26.2% in the post-test. The cognitive development of malnourished children in the intervention group improved compared to the control group (p < 0.001). This study revealed that home-based nutrition-focused food helps to enhance children’s cognitive development.

Trial registration: ctri@gov.in. CTRI/31/03/2017/008273 [Registered on: 31/03/2017].

## Introduction

Malnutrition in children is expressly detrimental. The damage to physical and cognitive development during early childhood is largely irretrievable^1^. A wide range of cognitive deficits has been reported in malnourished children^2^. Malnutrition is linked to suboptimal brain development, which has a negative impact on cognitive development, educational achievement, and economic productivity later in life^3^. During childhood, the maturation of specific brain areas is associated with the development of specific cognitive functions, such as language, reading, and memory. Synaptic density reaches a high level of brain growth during childhood age, and brain myelination (which regulates higher cognitive functions) persists well into adolescence^4^.

The genetic program that determines how each person's brain develops is modified by the environment, including nutrition^5^. Studies have demonstrated a link between good nutrition and optimal brain function^6,7^. Healthy nutrition supplementation plays a critical role in cell proliferation, DNA replication, neurotransmitter, and hormone metabolism and is an essential component of enzyme systems throughout the brain^8^. Early in childhood, the brain grows more quickly than the rest of the body, which could make it more susceptible to nutritional deficits^9^.

One of the factors affecting cognitive development is nutrition. Early childhood may be especially vulnerable to nutritional deficiencies due to the rapid brain growth throughout this period. Children who are malnourished have higher mortality and morbidity rates^10^. In preschoolers, impaired cognitive development is predictive of later school achievement^11^. Education increases one's human capital or the knowledge, resources, and skills that eventually determine health and well-being^12^. Poor motor and cognitive development, lower educational attainment, economic output, and a higher chance of developing metabolic illnesses can all have an impact on productivity during adulthood, which is a public health concern^13,14^. Studies have reported poor motor skills, adaptive behavior, language, and social skill development among malnourished children. Malnutrition is also linked to deficits in memory, visuomotor coordination, and social skills. Also, decreased IQ scores were associated with the severity of malnutrition^2^.

Despite the efforts of national and international obligations, the knowledge gaps of caregivers remain a cause for the nutritional status of children, and it affects physical growth and cognitive development. Considering this, the current study made an effort to give mothers comprehensive nutrition education to increase their understanding of and sensitise them to the prevention and management of malnutrition, change dietary habits, and enhance their children's cognitive development.

## Material and methods

### Study design and sample

**Figure 1:**
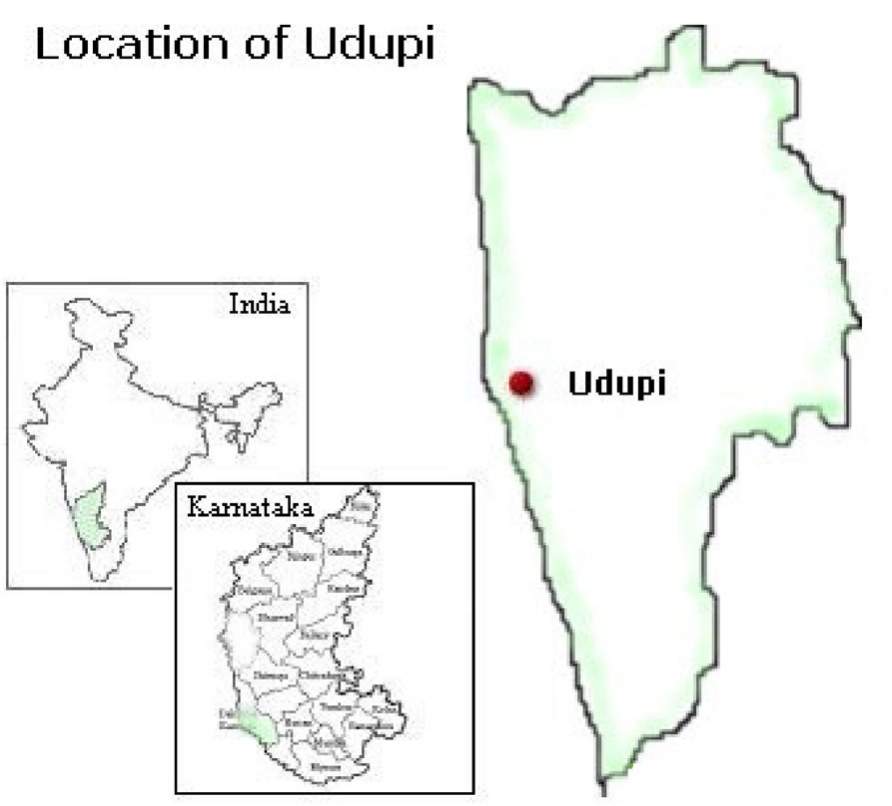
Area map of study location.

This study employed a cluster-randomised controlled trial design. The cluster of moderate and severe uncomplicated underweight preschool children between three and five years of age attending Anganwadi centers and their mothers in rural areas of Udupi district, Karnataka, were included in the study. Udupi district is a coastal part of Karnataka, India (Fig. 1).

#### Sample size

The sample size for the study was calculated considering 80% power, 5% level of significance, an attrition rate of 20%, a design effect of 1.5, an improvement of cognitive status in the standard of care of 30%, and 50% improvement in researcher intervention. Considering these, the required sample size was 230 for the intervention and control groups (115 in each group). Thus, 253 samples were included in the study from the selected clusters (127 + 126).

### Eligibility criteria for selection of sample

#### Inclusion criteria

The study included children between 3 and 5 years with moderate and severe underweight registered in Anganwadi centers and their mothers who could read/write/understand Kannada (local language) or English.

#### Exclusion criteria

Children aged between 3 and 5 years with known cases of systemic illness such as cardiac problems, renal problems etc., and not registered in Anganwadi centers were excluded from the study.

### Ethical approval

All methods were carried out in accordance with relevant guidelines and regulations. The study was approved by Kasturba Hospital Manipal, Institutional Ethics Committee and registered in the Clinical Trial Registry of India (CTRI/31/03/2017/008273). Reference number (REF/2016/08/012051) link http://ctri.nic.in/Clinicaltrials/pmaindet2.php?trialid=15547&EncHid=&userName=.

A detailed subject information sheet was given in the local language (Kannada) to all the mothers, and informed consent was obtained from each mother after explaining the purpose of the study and methodology and ensuring the confidentiality of the data obtained.

### Assessment of cognitive status

The cognitive status of the children was assessed with a standardized tool, “Cognitive development test for pre-scholars Scale” (PCDTP-p)^15^ for Indian children between 3 and 6 years of age by verbal and non-verbal tests. Dr Hema Pandey developed the tool to assess the cognitive development of Indian preschoolers. The tool includes six areas such as conceptual skills, information, comprehension, visual perception, memory, and object vocabulary. The tool had high validity, and the author reported test re-test reliability value is r = 0.95. The researcher also checked the current setting's content validity (CVI = 1) and reliability using the test re-test method (r = 1).

The assessor administered the test individually in an exclusively private room. Each child was given sufficient time to complete all the test items. Before the test, the child was made physically comfortable. Test results were recorded simultaneously on the scorecards. For each correct answer, one mark was awarded. The total score constituted the ‘raw’ scores of the subject (child). The total raw scores were converted to standard scores as per their age according to the table of norms and instructions provided along with the tool by the original authors of the tool. The following formula for conversion of raw score to standard score (Z-scores) was used. Z-Score = (X−M/SD): where X, score; M, mean of the raw score; SD, standard deviation of raw scores.

Based on the z score, the child’s cognitive development was interpreted (Table 1).

**Table 1:** Interpretation of the status of cognitive development.

z score range	Category	Cognitive development status
+ 2.01 and above	A	Extremely high development
+ 1.26 to + 2.00	B	High development
+ 0.51 to + 1.25	C	Above average development
− 0.50 to + 0.50	D	Average development
− 0.51 to − 1.25	E	Below average development
− 1.26 to − 2.00	F	Low development
− 2.01 and below	G	Extremely low development

### Randomization and procedure for data collection

**Figure 2:**
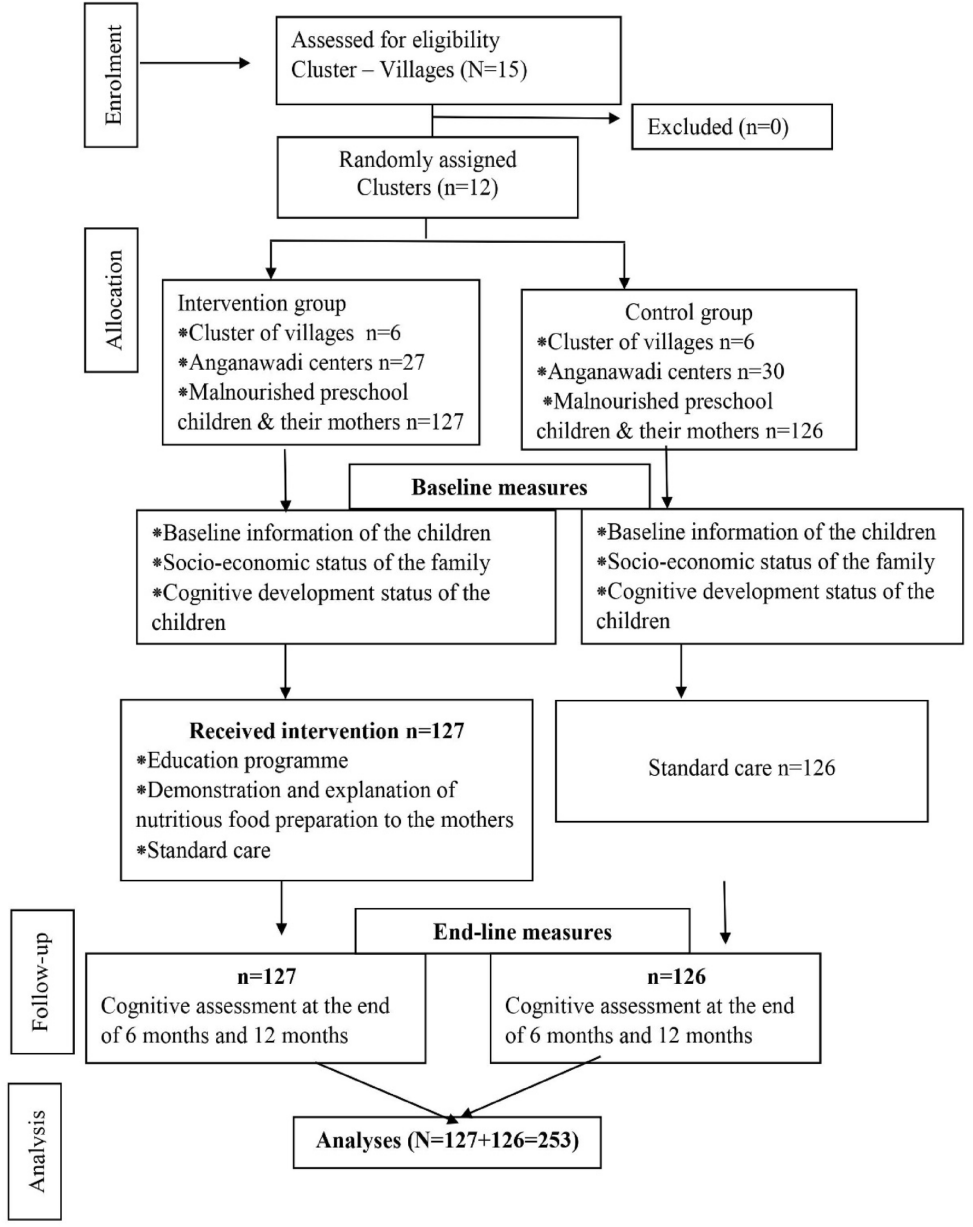
CONSORT (2010) flow diagram of the study.

Cluster randomization was adopted to select the villages. Twelve villages were chosen randomly for the study. Clusters of six villages were randomly assigned for the intervention, and six villages to the control group using the lottery method by the researcher. All Anganwadi centers belonging to allocated villages were included. Based on anthropometric indices, malnutrition status was identified. All malnourished children who met the inclusion criteria were included in the study. One hundred twenty-seven preschool children and their mothers from 27 Anganwadi centers in six villages were recruited for the intervention group, and 126 preschool children and their mothers from 30 Anganwadi centers in six villages were recruited for the control group (Fig. 2). Data were collected between March 2018 and May 2019. The researcher visited Anganwadi centers, and the weight and height of the children were measured. We evaluated the children’s nutritional status using weight-for-age and height-for-age indicators and graded the malnutrition level per NCHS’s new WHO child growth standards^16^. Moderate underweight was defined as a weight-for-age ratio of less than − 2 z score, severe underweight was defined as weight for age less than − 3 z score, and stunting was defined as a height-for-age − 2 z score. Moderately and severely underweight preschool children and their parents were selected for the study. The mothers of the malnourished children were met and informed about their children’s malnutrition, the purpose of the study, the data collection procedure, intervention, and follow-ups. The date and time for intervention at the Anganwadi center were set. The study’s nature was explained in detail, a subject information sheet was distributed in the local language, and informed consent was obtained.

After the mother signed the informed consent, baseline information was collected from the mother, and the researcher assessed the children’s cognitive development. With the help of power points, posters, and flipcharts, the mothers were educated on malnutrition, its causes, signs, and symptoms, how to identify malnutrition, the consequences of malnutrition on health and development in a child’s life, and a discussion was held on methods to prevent and manage malnutrition at home. A balanced diet, a malnourished child’s dietary requirements, and a sample diet menu plan for moderate and severe malnourished preschool children were also discussed.

One of the nutritious recipes, i.e., mixed vegetables and gram (paustic khichdi), was demonstrated. Additionally, the preparation of various healthy recipes rich in protein, energy, calcium, and iron were explained. The module on the Home-based management of malnutrition was given to the mothers, and they were advised to read it well and follow the practices presented in the module. Mothers were asked to prepare anyone nutritious food daily (or at least four times per week) and maintain a diary. They were also asked to send pictures of feeding the child with the recipe and clarify their doubts during the follow-up visit or telephonically. The monthly nutritional level assessment was carried out, and the findings are published^20^.

#### Follow-up and outcome measurement

Mothers’ queries were answered during the intervention phase, and suggestions were given when children had problems. Diary on nutritious diet preparation maintained by the mothers and dietary practices were checked during follow-up.

The outcome assessor performed an outcome assessment of cognitive development. The outcome assessor is a trained, qualified, graduated registered nurse and was blinded by the study group. The outcome measurement of cognitive development assessment was assessed at the end of six months (midline test) and 12 months (endline test) after the intervention in both groups.

### Data management and analysis

Data from 253 participants (Fig. 2) were pooled and analyzed using SPSS version 16. A chi-square test was carried out to test the homogeneity of baseline outcome variables among the intervention and control groups. The McNemar test and chi-square test were used to compare the cognitive improvement within and between the groups.

## Baseline characteristics of enrolled children

The mean age of children in the intervention group was 4.08 ± 0.71, and in the control group, it was 3.95 ± 0.68. A maximum number of children in both groups belonged to the family with a family income of less than 10,000 rupees/per month. The educational status of the parents (both mother and father) was lower in the intervention group than in the control group. Most of the fathers' occupations were reported as self-employed and mothers’ occupation were reported as small/self-business in both groups (Table 2). However, the comparison of baseline characteristics of both groups showed that, except for parents’ educational level, both groups were similar at baseline (Table 2).

**Table 2:** Baseline characteristics of enrolled children.

Variables	Intervention group Mean (SD)	Control group Mean (SD)	p-value
**Age of the child (in years)**	4.08 (0.71)	3.95 (0.68)	0.107
Gender of the child	f (%)	f (%)	
Male	59 (46.5)	49 (38.9)	0.138
Female	68 (53.5)	77 (61.1)	
**Income of the family/month** (in INR)			
5000–9999	74 (58.3)	61(48.4)	0.258
10,000–19,000	46 (36.2)	53 (42.1)	
20,000–50,000	7(5.5)	11 (8.7)	
> 50,000	0	1 (0.8)	
**Education of the father**			
Illiterate	24 (18.9)	4 (3.2)	0.0001
Primary	33 (26.0)	21 (6.7)	
Middle	19 (15.0)	26 (20.6)	
10th	37 (29.1)	46 (36.5)	
PUC	7 (5.5)	16 (12.7)	
Graduation	7 (5.5)	13 (10.3)	
**Education of the mother**			
Illiterate	18 (14.2)	2 (1.6)	0.0001
Primary	40 (3.5)	26 (2.6)	
Middle	22 (17.3)	14 (11.1)	
10th	28 (22.0)	44 (34.9)	
PUC	13 (10.2)	23 (18.3)	
Graduation	6 (4.7)	17 (13.4)	
**Occupation of the father**			
Self employed	101 (79.5)	90 (71.4)	0.194
Business	15 (11.9)	16 (12.7)	
Employed	11 (8.6)	20 (15.9)	
**Occupation of the mothe**r			
Self employed	62 (49.0)	44 (35.5)	0.075
Business	57 (45.0)	70 (55.5)	
Employed	8 (6.0)	12 (9)	

## Effect of intervention on cognitive development of malnourished preschool children

Table 3 demonstrates that most of the malnourished children in both groups at baseline had average and above-average cognitive development (intervention and control group). At baseline, 52% of children in the intervention group were classified as having average cognitive development; however, these numbers dropped by 14.2% in the midline test and 5.5% in the endline test and moved to the category of high and extremely high development. At the start of the trial, 44.4% of the children in the control group had average cognitive development; this number dropped to 28.6% at the midline and 26.2% at the endline test. Similarly, compared to the control group, more children in the intervention group scored high in the high and extremely high development categories on the midline and end-line tests (Table 3).

**Table 3:** Frequency and percentage distribution of preschool children in the intervention and control groups based on levels of cognitive development status at different points of assessment.

Cognitive development status	Intervention group (n = 127)			Control group (n = 126)		
	Baseline	Midline test	Endline test	Baseline	Midline test	Endline test
	f (%)	f (%)	f (%)	f (%)	f (%)	f (%)
High development	2 (1.6)	27 (21.3)	46 (36.2)	6 (4.8)	16 (12.7)	7 (5.6)
Above average	40 (31.5)	82 (64.6)	74 (58.3)	51 (40.5)	72 (57.1)	85 (67.5)
Average	66 (52.0)	18 (14.2)	7 ( 5.5)	56 (44.4)	36 (28.6)	33 (26.2)
Below average	18 (14.2)	0	0	12 (9.5)	2 (1.6)	1 (0.8)
Extremely low	1(0.8)	0	0	1 (0.8)	0	0

f, frequency, % percentage.

## Comparison of cognitive development status within the intervention group

Table 4 shows a significant difference in the baseline and midline test (Mc $\upchi$^2^_(df)_ = 78.89_(1)_, p =  < 0.001); baseline and endline test (Mc $\upchi$^2^_(df)_ = 53.94_(1)_, p =  < 0.001) of cognitive development scores. It is inferred that there was a significant improvement in the cognitive development scores of children in the intervention group over 12 months.

**Table 4:** Effect of intervention on cognitive development status in the intervention group.

							N = 127
Time points	HD	AAD	AD	BAD	LD	Mc $\upchi$^2^	p-value
Baseline	2	40	66	18	1		
Midline test	27	82	18	0	0	78.89_(1)_	< 0.001
Endline test	46	74	7	0	0	53.04_(1)_	< 0.001

HD, high development; AAD, above average development; AD, above average development; BAD, below average development; LD, low development.

## Comparison of pretest and post-test cognitive development status between the intervention and control groups

There was a significant improvement in children’s cognitive development from the baseline to the midline test. At six months of intervention (midline), 82 (64.6%) and 72 (57.1%) in both the intervention and the control group, respectively, were in the positive change category of cognitive development. The more positive category was higher (21.3%) in the intervention group than in the control group (12.7%), which was statistically significant (p = 0.006) (Table 5).

**Table 5:** Comparison of pretest and midline test cognitive development status between the intervention and control groups.

Cognitive development					N = 253	
	Intervention group (n= 127)		Control group ( n=126)		$\upchi$^2^_(df)_	p-value
	Frequency	Percentage	Frequency	Percentage		
More positive change	27	21.3	16	12.7	12.38_(3)_	0.006
Positive change	82	64.6	72	57.1		
Neutral	18	14.2	36	28.6		
Negative change	0	0	02	1.6		

Note: For the interpretation and understanding, the changes indicated are “neutral” as average cognitive development, above-average cognitive development as “positive change,” high development as “more positive change,” and below-average development as “negative change”.

In the intervention group, there was a significant increase in cognitive development from the pretest to the endline test, which was statistically significant (p < 0.001). In the midline and end-line tests, children in the intervention group moved from positive change (21.3%) to more positive change (36%). However, it was noticed that the more positive difference in the control group declined from the midline test (16, 12.7%) to the endline test (7, 5.6%). This explains a substantial difference in preschoolers' cognitive development between the intervention and control groups (Table 6).

**Table 6:** Comparison of pretest and endline test cognitive development status between the intervention and control groups.

Cognitive development					N = 253	
	Intervention group (n=127)		Control group (n=126)		$\upchi$^2^_(df)_	p-value
	Frequency	Percentage	Frequency	Percentage		
More positive change	46	36.2	7	5.6	52.88_(3)_	< 0.001
Positive change	74	58.3	85	67.5		
Neutral	7	5.5	33	26.2		
Negative change	0	0	1	0.8		

For interpretation and understanding, the changes indicated are “neutral” as average cognitive development, above-average cognitive development as “positive change,” high development as “more positive change,” and below-average development as “negative change”.

### Discussion

Various factors, including nutrition, influence the brain development. Nutritional deficiencies may have a significant impact on brain development^9^. In this study, we found that most of the preschoolers with malnutrition had poor cognitive development. We observed that 52% of malnourished preschool children in the intervention group and 44.4% in the control group had average cognitive development. We observed that, except for the motor speed and coordination test, malnourished children performed poorly on tasks requiring attention, working memory, learning and memory, and visuospatial ability. A study found that cognitive skills such as working memory, visual construction, learning, and memory in malnourished children were not developed as per age compared to adequately nourished children^2^, which supports the current study's findings.

In another study, children with malnutrition obtained lower social quotient scores than normal children. The effect was more noticeable in stunted, wasted children with poor communication and socialisation skills. Additionally, malnutrition had a significant relationship with immediate and delayed memory^17^. Higher absenteeism, falling sick, and poor school performance were found among Filipino children^18^.

Significant improvement in the cognitive development of preschool children was observed after implementing community-based nutrition intervention. The intervention developed and delivered was context-specific and affordable to the rural population^19^. Mothers exhibited enthusiasm in preparing varieties of nutritious food that were readily available and affordable to the family. We also observed an improvement in nutritional status among these children^20^. A community-based study from Indonesia reported improvement in the growth and development of children^19^. A systematic review of the effectiveness of early childhood interventions in promoting cognitive development revealed that the implementation of comprehensive programs on cognitive development was very effective. Interventions such as child-focused education and stimulation, parent-focused support, income supplementation, and nutrition and health interventions were effective in improving the cognitive development of children^21^. Food prepared by the mothers promote weight gain and cognitive development because the mothers can find food sources based on local availability, and children prefer local food prepared by the mothers^22^. Nutrition education intervention can improve the child’s growth parameters and overall development, including cognitive development. This intervention can be implemented within the integrated child development scheme (ICDS) framework so that mothers can provide nutritious diets to their children within their socioeconomic capabilities.

### Strength of the study

The outcome assessor, blinded to the study group, performed an outcome assessment of cognitive development. The outcome measurement, cognitive development, was assessed for a long duration, i.e., at the end of six months and 12 months after the intervention in both groups.

### Limitations of the study

The study included only preschool children and their mothers registered in the Anganwadi centres of selected villages of Udupi taluk. The intervention and control groups differed in terms of the parent’s educational level. The researcher could not control this as the study adopted cluster randomization.

## Conclusion

Malnutrition among children impacts their cognitive development. Thus, caregivers must be made aware of the effect of malnutrition on cognitive development so that they will be able to identify these deficits early and take corrective measures. Parenting and its related feeding practices in the first five years of life are closely related to stimulating children's overall growth and development.

## Data Availability

The data generated/or analysed during the current study are not publicly available due to confidentiality and ethical concerns. Still, they are available with the first author (Ms Ansuya) and/or corresponding author (Baby S. Nayak) on reasonable request.

## References

[1] UNICEF. Unicef for every child. Nutrition’s lifelong impact. UNICEF 2020 (accessed 18 June 2020); https://www.unicef.org/nutrition/index_lifelong-impact.html.

[2] Kar BR, Rao SL, Chandramouli BA (2008). Cognitive development in children with chronic protein energy malnutrition. J. Behav. Brain Funct..

[3] Leroy JL, Ruel M, Habicht JP, Frongillo EA (2014). Linear growth deficit continues to accumulate beyond the first 1000 days in low- and middle-income countries: Global evidence from 51 national surveys. J. Nutr..

[4] Toga AW, Thompson PM, Sowell ER (2006). Mapping brain maturation. Trends Neurosci..

[5] Bhatnagar S, Taneja S (2011). Zinc and cognitive development. Br. J. Nutr..

[6] Zeisel SH (2009). Importance of methyl donors during reproduction. Am. J. Clin. Nutr..

[7] De-Souza AS, Fernandes FS, Do-Carmo MG (2011). Effects of maternal malnutrition and postnatal nutritional rehabilitation on brain fatty acids, learning, and memory. Nutr. Rev..

[8] Zimmermann MB (2011). The role of iodine in human growth and development. Semin. Cell Dev. Biol..

[9] Benton D (2010). The influence of dietary status on the cognitive performance of children. Mol. Nutr. Food Res..

[10] Olofin I (2013). Associations of suboptimal growth with all-cause and cause-specific mortality in children under five years: A pooled analysis of ten prospective studies. PLoS ONE.

[11] Clark CA, Pritchard VE, Woodward LJ (2010). Preschool executive functioning abilities predict early mathematics achievement. Dev. Psychol..

[12] Engle PL (2010). INCAP studies of malnutrition and cognitive behavior. Food Nutr. Bull..

[13] Victoria CG (2008). Maternal and child undernutrition: Consequences for adult health and human capital. Lancet.

[14] Florence MD, Asbridge M, Veugelers PJ (2008). Diet quality and academic performance. J. Sch. Health..

[15] Pandey, H. Manual for Pandey’s cognitive development test for preschooler PCD TP-p. In *National Psychological Corporation, Bhargav Bhavan, Kacheri ghat, Agra, India* (1994).

[16] WHO child growth standards. Length/height-for-age, weight-for- age, weight- for-height and body mass index-for-age, methods and development (2006; accessed 13 July 2013).

[17] Upadhyaya SK, Agarwal KN, Agarwal DK (1989). Influence of malnutrition on social maturity, visual motor coordination and memory in rural school children. Indian J. Med. Res..

[18] Mendez MA, Adair LS (1999). Severity and timing of stunting in the first two years of life affect performance on cognitive tests in late childhood. J. Nutr..

[19] Susanto T (2019). Promoting children growth and development: A community-based cluster randomized controlled trial in rural areas of Indonesia. Public Health Nurs..

[20] Bengre A, Nayak BS, Unnikrishnan B (2023). Impact of a home-based nutritional intervention program on nutritional status of preschool children: A cluster randomized controlled trial. BMC Public Health.

[21] Rao, N. *et al*. Early childhood development and cognitive development in developing countries: A rigorous literature review. In *Department for International Development* (2014). https://www.researchgate.net/publication/321026156.

[22] Susanto T (2017). Local-food-based complementary feeding for the nutritional status of children ages 6–36 months in rural areas of Indonesia. Korean J. Pediatr..

